# Nature’s Medicine? The Associations of Organized Youth Sport, Unstructured Physical Activity, and Land-Use Recreation with Children’s Mental Health, Emotional Control, and Social Well-Being

**DOI:** 10.3390/ijerph22071012

**Published:** 2025-06-26

**Authors:** Natalie Stagnone, Katherine N. Alexander, Kat V. Adams, Travis Dorsch

**Affiliations:** 1 Elson S Floyd College of Medicine, Washington State University, Spokane, WA 99202, USA; 2 Department of Human Development and Family Studies, Utah State University, Logan, UT 84322, USA; k.alexander@usu.edu (K.N.A.); kat.adams@usu.edu (K.V.A.); travis.dorsch@usu.edu (T.D.)

**Keywords:** mental health, emotional control, social well-being, physical activity, youth sport

## Abstract

Physical activity is well-established as beneficial for children’s physical and mental health, yet limited research has explored how different activity types impact psychosocial outcomes. This is a cross-sectional study that examined associations between youth participation in organized sport, unstructured physical activity, and land-use recreation and their psychosocial health. Survey data were collected from 3246 caregivers of children in the Intermountain West region of the United States. Respondents reported on children’s physical activity engagement and psychosocial outcomes, including mental health, emotional control, and social well-being. All three activity types were significantly associated with improved psychosocial health (*p* < 0.001). Compared to organized sport, unstructured physical activity and land-use recreation were associated with significantly higher levels of mental health and emotional control (*p*_adj_ < 0.001), although with a small effect size (*η*^2^ < 0.006). No significant differences were observed in social well-being across activity types (*p* = 0.2928). When controlling for gender and community type, distinct psychosocial benefits emerged between activity types. These findings suggest that, while all forms of physical activity support psychosocial development, unstructured and nature-based activities may offer particular advantages. Findings highlight the importance of promoting diverse forms of physical activity and considering individual and contextual factors in maximizing their psychosocial benefits.

## 1. Introduction

Physical activity can benefit a person’s physical health and psychosocial wellbeing, and these benefits can be especially true for youth [1]. The short-term benefits of physical activity for children are far-reaching, including higher fitness levels, lower body fat, improved academic performance, improved cognition, and decreased symptoms of depression [2,3]. It is known that physical activity, generally, can benefit multiple aspects of youth’s psychosocial wellbeing, including mental health, emotional control and social wellbeing, but it is unclear what forms of physical activity may be of most benefit. In the long-term, engaging in appropriate levels of physical activity as a child and adolescent improves the likelihood of maintaining lifelong healthy habits [2]. Continuing healthy habits reduces a person’s likelihood of developing some modifiable chronic diseases, such as type 2 diabetes mellitus, cardiovascular disease, and certain cancers [2,3].

Currently, the US Department of Health and Human Services recommends children and adolescents engage in 60 min of aerobic, muscle-strengthening, or bone strengthening exercises every day [3]. However, recent estimates suggest that just one in four youth in the United States are meeting these guidelines [3,4]. As children age, their physical activity tends to decrease, a trend that is especially pronounced for girls and those in rural areas [5,6]. Further, youth are increasingly experiencing more mental health concerns, which are at least partially explained by decreased amounts of physical activity and increasing amounts of screen time and sedentary behaviors [7,8]. Therefore, it is increasingly important to understand what forms of physical activity could be most influential on different aspects of a youth’s psychosocial wellbeing.

During the recent COVID-19 pandemic, numerous restrictions were imposed that limited children’s opportunities for physical activity (e.g., the closure of exercise facilities, the cancelation of youth sports, virtual schooling). This led to even fewer youth meeting the physical activity guidelines in the United States and abroad [9,10,11]. Now, five years after the beginning of the pandemic, there are calls to adjust how scholars and practitioners design and deliver physical activity spaces and opportunities, including ensuring more equitable access during childhood and adolescence [12]. Given these gaps, the current study is designed to learn more about the psychosocial benefits of different physical activities and whether there are specific benefits for these varied physical activity types in the post-COVID era. Differences based on gender and community type will also be explored, given that there are known disparities in physical activity involvement across these groups [13,14].

### 1.1. Physical Activity Contexts

Physical activity includes “any bodily movement produced by skeletal muscles that results in energy expenditure” [15] (p. 126). In the United States, youth participate in many forms of physical activity, falling on a continuum from more to less structured. A simple classification system for these contexts includes organized youth sports (i.e., physical activity that is typically directed by adults and involves rules and formal practice and competition), unstructured physical activity (i.e., play opportunities that are child-led and generally occur without a defined purpose or outcome), and land-use recreation (i.e., use of public or private spaces for the enjoyment of their natural, scenic, or aesthetic qualities and for compatible recreational activities).

Youth Sports. Youth sports are defined as physical activity that is typically directed by adults and involves rules and formal practice and competition [1]. As many as 54.1% of children participate in organized youth sports each year, with team sports (e.g., basketball, soccer, baseball) being the most popular in the United States [16,17]. The most obvious benefit of participation is that youth sports provide opportunities to engage in regular, structured physical activity under the guidance of (presumably) trained adults [18]. Yet, the benefits of this participation go beyond physical activity, with a body of research showing the potential benefits of youth sports on children’s and adolescents’ mental health [1,19], including psychosocial benefits, education and lifelong learning, crime reduction, reduction in anti-social behaviors, and active citizenship [1,18,19]. Children who participate in organized youth sports in communities, schools, or clubs commonly report fewer depressive symptoms, less anxiety, better self-esteem, and more social interactions compared to non-participants and find that sports support friendship development [1,18]. In youth sports, these benefits can be lifelong, especially when “fun” and psychological well-being are emphasized alongside physical activity [18].

However, as noted by Eigenschenk et al. (2019), sport is not an inherently positive experience, and issues of accessibility are often involved in these environments [19]. Gender stereotypes are often prevalent in youth sports, with certain sports being deemed “appropriate” for females or males (i.e., football and golf are perceived to be male sports, whereas gymnastics and dance are perceived to be female sports) [20]. Female athletes face additional gendered barriers to youth sport participation, including fewer positive role models, fewer opportunities in general, and fear of social stigma [20]. These barriers may be mediated by internalized negative beliefs of female stereotypes, beliefs of what the expectations are for their age level, or low expectations of sport performance [20]. More broadly, there are several other negative aspects associated with youth sports involvement, including increased risk of injury, financial burden for the family, pressure to specialize in one sport, challenges in finding a good coach, and attrition as children age [18].

The COVID-19 pandemic exacerbated many of the disparities that exist in youth sports [21,22]. While most of the prior research on physical activity and mental health was conducted prior to the pandemic, more recent research has highlighted the impact of COVID-19. Importantly, during the pandemic, restrictions in youth sports particularly affected female youth athletes, who experienced greater anxiety and depression symptoms [23]. Parents residing in urban neighborhoods were also more concerned about the barriers to returning to sport [21]. Now, post-pandemic, there have been calls for youth sports to become more accessible (especially in rural or underserved areas), consult with athletes about their needs, emphasize fun, social connections, collaboration and being active, and empower young people (giving them a sense of autonomy) [12].

Unstructured Physical Activity. Play opportunities that are child-led and that generally do not have a defined purpose or outcome are often referred to as unstructured physical activity and are an important part of children’s development [24]. Unstructured physical activities can include free time spent during school at recess time, running through the neighborhood park, or playing soccer with peers. There are many psychosocial benefits to unstructured physical activity, including promoting increased physical activity, social engagement, emotional well-being, self-determination, independence, and creativity [24,25]. Research on the various contexts of physical activity has been somewhat equivocal, as there is evidence to suggest structured physical activity may be a more powerful predictor of positive outcomes for youth. Indeed, Subramanian and colleagues (2015)—in a randomized control study—found that adolescents who participated in structured physical activity (rather than unstructured physical activity) had better improvements in their cognitive function, although there were cognitive improvements in both groups [26]. Research suggests that one important benefit to unstructured forms of physical activity is that children and adolescents are more likely to continue to pursue unstructured forms of physical activity outside of school and further into adulthood; this is a trend not seen in more structured forms of physical activity [25].

Most families encourage their children to participate in physical activity via play opportunities [27]. Despite this, in a study conducted prior to the COVID-19 pandemic, it was found that children are currently participating in less unstructured physical activity, opting for indoor screen time or structured activities (e.g., organized sport) when they are outdoors [28]. Decreased unstructured physical activity may be related to a child’s decreased ability to self-regulate, leading to aggression and impaired social interaction [24]. Notably, among children and adolescents, those who engage in unstructured forms of physical activity may do so because they are more motivated to succeed academically, leading to less time or desire to engage in organized sports leagues or teams [5]. Gender can also impact a child’s decision to participate in unstructured physical activity. Youth who identify as female tend to participate in lower levels of physical activity, with this group experiencing a greater decline in physical activity during the initial part of the COVID-19 pandemic [13]. There has been little research conducted to explore the impact of community type on unstructured physical activity, with only one study finding that an increased number of “physical activity resources” in close geographic proximity was associated with increased physical activity among high school females [29]. Although more work is needed on this subject, it could be inferred that individuals in rural areas may have fewer opportunities to engage in unstructured physical activity due to fewer resources within a close geographic proximity.

There were mixed findings in regard to trends in unstructured physical activity during the COVID-19 pandemic. Overall, youth physical activity was found to decrease; however, this decrease was likely due to decreases in structured physical activity opportunities (i.e., youth sports) [9]. Two studies reported that there was an increase in unstructured physical activity during the pandemic, potentially leading to increased physical activity in youth [30,31]. Given the lasting impact of the pandemic on youth and families, more research is needed to evaluate unstructured physical activity and its potential specific benefit for children and adolescents, including new trends in participation and attrition.

Land-Use Recreation. Land-use recreation is defined as the use of public or private spaces for the enjoyment of their natural, scenic, or aesthetic qualities and for compatible recreational activities. Examples of land-use recreation include hiking, paddle boarding, rafting, skiing, or mountain biking, and it is publicly perceived as promoting healthy outcomes for youth. Compared to indoor physical activity, outdoor physical activity is hypothesized to support positive emotions, tranquility, restoration, and motivation [32]. Although a majority of studies are conducted on college or university campuses, these findings suggest that outdoor physical activity supports greater feelings of positive engagement, revitalization, energy and decreased anger, tension, and depression [33]. Furthermore, a growing body of evidence suggests that exposure to natural environments can improve attention and decrease stress in children, among other possible benefits [34,35,36], and these benefits may or may not be obtained through specific nature-based interventions [37]. These benefits for children are emphasized in several reports that call for pediatricians and other pediatric healthcare providers to recommend outdoor physical activity as a way to counteract a variety of child health problems [34,36]. Nonetheless, there is a lot of promise for the benefits of outdoor physical activity and more research is needed to confirm its relative benefits compared with other categories of physical activities.

Differences in participation in land-use recreation appear to exist across gender and community type. According to one report, it is estimated that 54% of individuals over six years old performed at least one outdoor activity that year in 2022 [38]. Gender differences in patterns of recreation are also apparent, with adults identifying as female being less inclined to participate in land-use recreation activities alone, less likely to engage in nature-based recreation (despite stating that they enjoy it more relative to men), and less likely to participate in certain outdoor activities, such as hunting or fishing [39]. Community type (urban, suburban, or rural) also impacts one’s ability to participate in land-use recreation. According to a national study, urban communities (regardless of income) generally have more parks/green spaces available, while rural areas may have more natural spaces but face barriers like limited access to facilities and organized programs [14]. Broadly, those who are closer in proximity to a park or other outdoor recreational space are more likely to engage in higher levels of physical activity [40], although this relationship is complex, due to other social factors that can impact the use of these green spaces (e.g., socioeconomic status) [14]. Regardless, the extant literature indicates that gender and community type likely play important roles in participation in land-use recreation activities.

During the COVID-19 pandemic, there were increases in land-use recreation and outdoor activity, and, now five years later, use of these spaces continues at pre-pandemic levels [41]. For youth, there are little data on the impact of COVID-19 on trends in land-use recreation. However, one study did find that youth who participated in outdoor activities, such as bicycling, going for walks or runs, or swimming, during the pandemic saw improved resilience and smaller declines in their social well-being compared to youth not participating in such activities [42]. Further research is needed to evaluate the specific trends seen in youth land-use recreation, including trends distinguished by gender and community type.

### 1.2. Physical Activity Outcomes

Mental Health. Physical activity has the potential to improve a child’s mental health [19,34,43,44]. Mental health is challenging to define but includes a person’s social, emotional and psychological well-being [45]. Across the United States, mental health conditions, such as anxiety and depression, have been on the rise in youth and were exacerbated by the COVID-19 pandemic [23,46,47]. Generally, physical activity of any kind is thought to reduce anxiety and depressive symptoms and to improve cognitive function, especially in children and adolescents [2,43,48]. Increased physical activity is more strongly associated with decreased symptoms of depression, while there is less evidence to support the association of increased physical activity with decreased anxiety [43,48]. It is also more commonly assumed that being outdoors (rather than exercising inside) gives further benefit to one’s mental health, but there is limited evidence to support this claim [19,32]. Taken altogether, physical activity is positively associated with improved mental health in children and adolescents, but more research is needed to determine what types of physical activity may be most beneficial.

Emotional Control. Physical activity also has the potential to improve a child’s emotional control [19,49,50]. Emotional control is the ability to control and regulate affect and feelings [51]. On the whole, research suggests that exercise does not impact adults’ ability to regulate their sadness or anger in the short-term but that long-term physical activity can improve their abilities to regulate their emotions and cope with stress [19,52,53,54]. Further, physical activity can benefit a child’s executive function, which includes aspects of emotional control [49]. In children with attention deficit hyperactivity disorder (ADHD), recurring “chronic” physical activity completed for over 30 min per session can improve these children’s emotional control, as well as having other cognitive and behavioral benefits [44,50]. However, it is unclear how these trends may be dependent on the type of physical activity.

Social Well-Being. Finally, physical activity has the potential to improve a child’s social well-being [1,48]. Social well-being is an individual’s feelings of positive connection and support from others as well as their general inclusion within society [45]. Although there remains limited research into how social well-being is influenced by physical activity, social well-being may mediate the positive mental health benefits seen with physical activity [1,48]. It is unclear whether this differs based on the type of physical activity involvement, but these positive effects may be most prominent in activities that involve groups or teams, that can encourage pro-social behaviors among youth [1,19]. Importantly, social well-being benefits may be most profound for youth participating in outdoor recreation settings [42].

### 1.3. The Present Study

While prior research has established that organized youth sport, unstructured physical activity, and land-use recreation benefit youth’s mental health, emotional control or social well-being, no research has directly compared different forms of physical activity and their associated psychosocial wellbeing outcomes after the start of the COVID-19 pandemic [1,19,24,25,32,36]. To address this important gap, the present study was designed to directly compare each of these psychosocial outcomes across youth sports, unstructured physical activity, and land-use recreation settings as a way to explore potential differences in benefits across these varied activity domains. Given literature highlighting potential differences in experiences across gender and community type, additional analyses to explore potential differences in these associations were also conducted. Considering robust findings from the contemporary literature showing the benefits of outdoor recreation [32,34], we hypothesize that land-use recreation will be associated with significantly higher mental health, emotional control, and social well-being scores when compared to organized youth sport and unstructured physical activity. We also hypothesize that differences will exist across activity type and outcomes by gender and by community type, with females demonstrating significantly higher psychosocial benefits from land-use recreation compared to males, and urban areas demonstrating significantly higher psychosocial benefits from organized youth sport compared to suburban and rural areas.

## 2. Materials and Methods

### 2.1. Participants

The current study is part of a large mixed-methods, cross-sectional study that was designed to evaluate organized youth sport, unstructured physical activity, and land-use recreation in the Intermountain West region of the United States. The study was approved by the Institutional Review Board of Utah State University and adhered to APA 7th edition ethical standards. Survey respondents were parents or caretakers of youth aged 6 to 18 in Colorado, New Mexico, Utah, and Wyoming. Respondents were English-speaking residents of the four states. No health-related information was captured in survey responses. Respondents were initially recruited via Qualtrics panel and completed online questionnaires. They received USD 5 for their participation. A Qualtrics survey management team screened the responses for completeness and ensured that established sociodemographic quotas were met. Data collection via Qualtrics has been considered reliable and valid, with prior research indicating that Qualtrics serves as a solid method for data collection [55]. Data were collected between 25 July and 7 November 2022.

A total of 3519 adult respondents completed the questionnaire and reflected on their child’s physical activity involvement. Two hundred and seventy-three respondents were excluded for indicating that their child was under 6 or over 18 years old in an initial demographic screener. The average age of adult respondents was 39.37 years (*SD* = 10.63, range = 18–78). Of the respondents, 68.85% identified as male, 29.98% as female, 0.71% as nonbinary, 0.18% as “prefer not to say,” and 0.28% as “other.” Most respondents were married (55.18%), with an average household income of USD 89,938.49. Of the respondents, 71.35% identified as White, 12.01% as Hispanic, Latino, or Spanish origin, 8.38% as Black or African American, 3.79% as Asian, 1.69% as Multiracial, 1.39% as American Indian or Alaskan Native, 0.28% as Native Hawaiian or Pacific Islander, 0.62% as “prefer not to say,” and 0.49% as “other.” Most respondents worked full-time (61.21%), followed by 7.02% self-employed, 6.87% homemakers, 6.87% working part-time, 4.87% being unable to work, 3.76% being retired, 3.67% being out of work, 1.82% preferring not to answer, and 1.05% as “other.” Lastly, 40.45% of respondents identified as living in an urban neighborhood, 39.49% living in a suburban neighborhood, and 20.06% in a rural neighborhood.

### 2.2. Measures

Respondents were asked to indicate the number of children in their household, followed by the number of children who participated in organized youth sport, unstructured physical activity and land-use recreation. When respondents indicated that their child(ren) participated in one or more of these contexts, they were asked to complete a series of questions about one child who participated most regularly or was most committed to participating in that activity. For each category, respondents were asked to rate their child’s mental health, emotional control, and social well-being on a 1 to 5 Likert scale (i.e., Reduced GREATLY or 1, Reduced SOMEWHAT or 2, Not Changed or 3, Enhanced SOMEWHAT or 4, and Enhanced GREATLY or 5) as a result of their participation in that category. All measures were single-item, study-designed measures constructed collaboratively with the research team.

### 2.3. Data Analysis

Data were stored on Box.com, an encrypted, cloud-based storage system. For each category of physical activity, the researchers evaluated and compared the psychological benefits (mental health, emotional control, and social well-being). The Shapiro–Wilk test was used to determine whether the sample was normally distributed [56]. The Shapiro–Wilk test was statistically significant (*p* < 0.0001) across all physical activity contexts, justifying the use of Kruskal–Wallis Tests. Kruskal–Wallis rank-sum tests are useful for making comparisons with non-normal data [57] and were used to examine statistically significant differences in these psychological outcomes across each category of physical activity. Subsequent pairwise comparisons for any significant results between psychological outcomes using Dunn’s tests for stochastic dominance with Bonferroni corrections were deemed appropriate, given that these are more robust to multiple pairwise comparisons and non-normality [58]. Kruskal–Wallis tests, with follow-up Dunn’s tests for significant results were also completed across gender and community type. All descriptive and inferential statistics were conducted in R, version 12.0+467 [59].

## 3. Results

Across the parent respondents, 54.68% indicated that they had at least one child who participated in one or more organized youth sports, 57.18% had at least one child participating in unstructured physical activity (PA), and 49.41% had at least one child participating in land-use recreation. Respondents indicated that 69.83% percent of their children participated in organized youth sports, 75.92% participated in unstructured physical activity, and 68.42% participated in land-use recreation. Within each activity, respondents were asked about the child they were evaluating and completed those questions for only one child. For organized youth sports, the average child age was 11.67 years (*SD* = 3.31, range = 6–18 years, *n* = 1258) and 62.80% percent were male (36.01% female, 0.95% nonbinary, 0.24% “prefer not to answer,” and 0.00% “other”). For unstructured physical activity, the average child age was 11.41 years (*SD* = 3.23, range = 6–18 years, *n* = 1220) and 53.36% percent were male (45.41% female, 1.23% nonbinary, 0.00% “prefer not to answer,” and 0.07% “other”). For land-use recreation, the average child age was 11.79 years (*SD* = 3.16, range = 6–18 years, *n* = 1117) and 58.28% percent were male (40.64% female, 0.72% nonbinary, 0.18% “prefer not to say,” and 0.18% “other”). Cohen’s *d* values for age ranged from 0.13 to 0.19, indicating a small effect size, while Cohen’s *d* values for gender ranged from 0.22 to 0.54, suggesting a moderate effect size. Percentage distributions of responses on Likert scales are in Table A1.

### 3.1. Overall Associations of Physical Activity Type and Outcomes

All three physical activity types were shown to benefit youth mental health, emotional control, and social well-being (see Table 1 for comparisons of means and standard deviations of outcomes by activity type). The Kruskal–Wallis test revealed a significant difference in mental health scores across activity types (χ^2^ = 21.38, *p* < 0.001), although the effect size was very small (*η*^2^ = 0.006). Dunn’s tests with Bonferroni corrections showed that youth sports mental health scores were significantly different from those for both unstructured physical activity (*p* < 0.001) and land-use recreation (*p* < 0.001). No significant difference was observed between unstructured physical activity and land-use recreation (*p* = 0.462). Similarly, the Kruskal–Wallis test revealed a significant difference in emotional control scores across activity types (χ^2^ = 18.328, *p* < 0.001, *η*^2^ = 0.005). Post-hoc Dunn’s tests with Bonferroni corrections suggested that children participating in organized youth sport showed a significantly lower emotional control score compared to both unstructured physical activity (*p* = 0.0003) and land-use recreation (*p* = 0.0004), with no significant difference between unstructured physical activity and land-use recreation (*p* = 0.486). Finally, the Kruskal–Wallis test revealed no significant differences in social well-being scores between the three physical activity types (χ^2^ = 2.4562, *p* = 0.2928, *η*^2^ = 0). See Table 2 for full results of the Kruskal–Wallis and Dunn’s tests.

### 3.2. Associations by Gender

Some differences in means and standard deviations were reported across gender, but all responses tended to skew towards positive endorsements of outcomes (see Table 3 for means and standard deviations for outcome and activity type, split by gender). Youth who identified as male and female both showed the same pattern as described above: youth sports were associated with significant differences in mental health scores and emotional control scores compared to unstructured physical activity and land-use recreation. Interestingly, there was a significant difference in social well-being scores between unstructured physical activity and land-use recreation in youth who identified as female (see Table 4 for full results of Kruskal–Wallis and Dunn’s test by gender).

### 3.3. Associations by Community Type

Participants across all community types tended to respond positively when reflecting on physical activities and outcomes, with some differences existing across groups and activity types (see Table 5 for means and standard deviations for outcome and activity type, split by community type). In urban and rural areas, participants in land-use recreation differed in mental health scores compared to organized youth sport and unstructured physical activity. Additionally, in rural areas, participants in land-use recreation differed in emotional control scores compared to organized youth sport and unstructured physical activity. In suburban areas, participants in unstructured physical activity differed from land-use recreation in both mental health scores and emotional control. Organized youth sport also differed from land-use recreation for social well-being scores in suburban areas. See Table 6 for full results of Kruskal–Wallis and Dunn’s test by community type.

## 4. Discussion

The present study was designed to examine the ways in which children’s physical activity engagement is associated with their psychosocial health. Unsurprisingly, all three physical activity categories positively impacted children’s mental health, emotional control, and social well-being. However, this study adds to the literature to indicate that certain forms of physical activity may have better psychosocial outcomes in some young people. Most significantly, our results suggest that organized youth sport involvement was perceived to be associated with significantly lower mental health and emotional control scores than unstructured physical activity and land-use recreation.

These findings partially align with study hypotheses that land-use recreation would be most strongly associated with positive mental health, emotional control, and social well-being outcomes. Prior research has shown many benefits of outdoor recreation, including reducing anger, tension, and depression as well as promoting more positive emotions, tranquility, and motivation [32,33]. Therefore, it is unsurprising that youth mental health and emotional control would benefit from land-use recreation (although with a very small effect size).

More interesting, perhaps, is the indication that unstructured physical activity has more perceived benefit for a child’s mental health and emotional control than organized youth sport. This is not fully aligned with current emerging literature comparing structured and unstructured forms of physical activity. For example, adolescents in one study who participated in structured physical activity (rather than unstructured physical activity) had better improvements in their cognitive function, although there were cognitive improvements in both groups [26]. This seems to suggest that there would be more benefits to structured physical activity (i.e., organized youth sport), which contrasts with our study findings. It may be that unstructured physical activity is especially important post-COVID and with decreasing opportunities for children to be independent [10]. It may also be that our study design is related to this unexpected finding: parents may perceive these psychosocial benefits (self-determination, autonomy, independence, etc.) as more beneficial to children in relation to unstructured physical activity, despite other studies that highlight child-reported benefits and other benefits of different physical activity types. Regardless, it is apparent from research and our own current findings that unstructured physical activity is beneficial and improves children’s psychosocial health, likely via increased self-determination, independence, and positive social engagement [24,25].

Results may also indicate that parents perceive organized youth sport to have less benefit than land-use recreation and unstructured physical activity. While a large part of the literature on organized youth sport indicates largely positive outcomes, there are inherent possibilities for negative outcomes [1,18,19]. Some children may experience gender stereotypes or increased risk of injury, and families may find that youth sports are a financial burden [18]. It is possible that these negative aspects of organized youth sport may counteract some of the benefits and be responsible for the trends observed in this study. Overall, findings highlight the potential for greater perceived mental health and emotional control when participating in unstructured physical activity and land-use recreation compared to organized youth sport, although effect sizes were small.

Interestingly, analyses highlight no significant differences in social well-being across the three physical activity types. Prior research has suggested that social well-being is a mediator for positive mental health benefits seen with physical activity [1,48]. While we did not directly compare psychosocial benefits, our results extend present knowledge by highlighting distinct social well-being benefits in these three physical activity contexts. Group or team activities have been theorized to benefit children’s social well-being most [1,19], yet our research appears to contrast with this expected finding, instead suggesting that there is no difference between social well-being benefits in individual versus group physical activities.

Lastly, this study took place in 2022, immediately after the COVID-19 pandemic. It is known that the pandemic changed the ways in which youth participated in physical activity [7,8,21]. While this study is novel in many respects, the more long-term impacts of the pandemic on trends remains to be seen. The current study remains pivotal in beginning to question how modern societal changes impact trends in physical activity, including positing inquiries about whether there have been significant changes in organized youth sport (making it less beneficial for children’s psychosocial health), if more children are finding benefits from land-use recreation than before the initial pandemic onset, or if youth are facing new and unique challenges that have not been captured in previous surveys. This current study may serve as a catalyst to reconsider potential unique benefits and challenges of the different physical activity contexts in the post-COVID era. Indeed, it is plausible that, even in 2022, organized youth sport settings were more restrictive (more mask wearing, social distancing, etc.) compared to unstructured physical activity and land-use recreation. This provides a possible explanation for why organized youth sport did not benefit children’s mental health and emotional control compared to the other physical activity types.

Overall, our findings bolster the idea that organized youth sport, unstructured physical activity, and land-use recreation all have perceived benefits for children’s mental health, emotional control, and social well-being [2,43]. Our findings also indicate potential for greater perceived mental health and emotional control when participating in unstructured physical activity and land-use recreation compared to organized youth sport, despite smaller effect sizes. As the dynamic physical activity landscape continues to normalize post-pandemic, we align with previous research to support an emphasis on fun, social connections, collaboration, activity, and empowerment of young people through organized youth sport and other physical activities [12].

### 4.1. Gender

Tests of group differences highlighted that males and females showed similar patterns, compared to without controlling for gender. Prior research on organized youth suggests that gender stereotypes continue to perpetrate the experiences of female youth, including female youth having fewer opportunities, fewer role models, and more social stigma [20]. It is interesting, then, that the current study seems to show that organized youth sport decreases both male and female youth’s mental health and emotional control, with no significant difference in females’ mental health between organized youth sport and land-use recreation.

Importantly, in females, there was also a significant difference in social well-being scores when comparing between unstructured physical activity and land-use recreation. This indicates that there is an increase in social well-being scores for females when participating in unstructured physical activity compared to land-use recreation. There was no significant difference for males or when not controlling for gender. Potential reasons for this unique result are that women tend to not want to participate in land-use recreation alone, are less likely to participate in certain land-use recreation activities (i.e., hunting or fishing), and tend to engage in nature-based activities less frequently than males [39]. These reasons suggest larger cultural and societal influences on social well-being than just the physical activity contexts. For children, these influences may lead them to find less comfort when participating in land-use recreation, but when they do, they find the same mental health and emotional control benefits. When considering social well-being, it is important to consider the nuances of gender and gendered expectations.

### 4.2. Community Type

Differences in psychosocial health benefits based on community types were additionally apparent and unique compared to previous results. For urban communities, the only significant finding in the current study was that organized youth sport was associated with significantly lower mental health scores when compared to unstructured physical activity and land-use recreation—contradicting our original study hypothesis. Previous research has found that urban environments tend to have more parks/green spaces available and that closer proximity to these spaces makes individuals more likely to engage in higher levels of physical activity [14,40]. This research provides a possible explanation for our study results: higher access to parks/green spaces is related to positive mental health benefits in youth who use these spaces to undertake unstructured physical activity and land-use recreation. Alternatively, the small effect sizes seen in our study could suggest that this finding is less pragmatic or that youth in urban areas see psychosocial benefits in all physical activity contexts. Future studies should explore whether greater access to green spaces/parks can mediate mental health benefits in youth participating in physical activity in these spaces.

Similarly, youth living in suburban communities had unique psychosocial benefits. Comparing organized youth sport to unstructured physical activity, unstructured physical activity showed significant and larger mental health (*p adj^a^* = 0.009) and emotional control (*p adj^a^* = 0.005) scores. Comparing organized youth sport to land-use recreation, the only significant result was in social well-being (*p adj^a^* = 0.046), with organized youth sport having significantly higher social well-being scores (*M* = 4.01, *SD* = 1.03) compared to land-use recreation (*M* = 3.85, *SD* = 1.10). Our research suggests that the mental health and emotional control of youth in suburban communities may benefit most from unstructured physical activity, whereas their social well-being may benefit most from organized youth sport. These results are particularly unique compared to overall tests of the relationship between physical activity type and psychosocial outcome. It is intriguing, therefore, to consider how suburban youth may have different experiences, positive and negative, when participating in physical activity. Little research has been performed considering suburban youth and influences on their physical activity types. Future research should evaluate suburban youth and their unique experiences participating in physical activity, especially in unstructured settings. While land-use recreation still positively impacted on children’s psychosocial health, this research suggests that children in suburban communities are perceived to benefit less from land-use recreation.

Within rural communities, our results again underscore the potential unique psychosocial effects of different physical activity types for residents of these communities. Prior research has described how rural communities have more access to natural areas but face challenges in participating in certain physical activity types because of longer distances to travel and limited access to facilities and organized programs [40]. These barriers to access may explain why organized youth sport was associated with significantly lower mental health scores compared to land-use recreation and significantly lower emotional control scores compared to unstructured physical activity and land-use recreation. Broadly speaking, this raises the question of how access to resources (e.g., sports teams) might impact psychosocial outcomes for youth, given that there was no difference in social well-being among the different physical activity contexts in the current study. More research is surely needed to investigate potential reasons for this unique psychosocial composition of children in rural areas.

### 4.3. Limitations and Future Directions

Despite the contributions made by this study, it has limitations that should be acknowledged and discussed. First, the sample was drawn exclusively from a four-state region of the Intermountain West United States at one point in time, potentially limiting certain aspects of generalizability. Given the geographical, historical, and sociodemographic characteristics of this region, this study provides a unique case study into children’s organized youth sport, unstructured physical activity, and land-use recreation. This study was also cross-sectional, only providing information on a brief snippet in time in the post-COVID era. In light of this, scholars should avoid drawing broad conclusions from this work. Instead, contextualized learnings can be used to help design future work with other diverse populations living in the United States or the world.

A second limitation has to do with the blurred lines between organized youth sport, unstructured physical activity, and land-use recreation. Although the current study operationalized these term, there is much overlap between them. This is especially true in this region, where sports like mountain biking, trail running, and snow skiing are prevalent in schools and communities (i.e., organized youth sports) but are also undertaken by individuals and families as a form of leisure (land-use recreation). Additionally, children likely participate in multiple forms of physical activity and could have been included in multiple physical activity types within this study. The survey was designed to ask the respondent to report on what types of physical activities their child participates in and to describe trends only for their child that participates the most in that given activity type. Future studies could be designed to better understand the unique aspects of these three arenas of participation as well as the ways in which the boundaries are blurred.

Next, there are limitations to collecting data using a survey. First, the survey utilized single-item measures as an indicator, which limits our scope of understanding around psychosocial benefits. Future research should consider implementing subjective and objective measures of wellbeing. Next, there is also the potential for social desirability bias, whereby respondents completed the survey based on making themselves appear in a positive light. Future research should consider using mixed methods or alternative sampling strategies to capture a more diverse and representative population and to validate self-reported responses.

A final limitation has to do with the appropriateness of the respondents to our survey. Although the sample was robust and nationally representative, there are inherent risks to asking parents to report on the uniquely held experiences of their children. Indeed, privileging parents’ perceptions of children’s mental health, emotional control, and social well-being over their child’s leaves open the possibility of response bias. Although parent responses likely serve as a relative proxy in most cases, future work should be designed to directly assess children’s perceptions of these same variables. Moreover, qualitative research should be utilized to better understand the mechanisms by which organized youth sport, unstructured physical activity, and land-use recreation influence children’s mental health, emotional control, and social well-being.

## 5. Conclusions

The U.S. Department of Health and Human Services recommends that children and adolescents engage in at least 60 min of daily aerobic, muscle-strengthening, or bone-strengthening activity; however, only one in four youth in the United States currently meet these guidelines [3]. Given the growing mental health crisis among youth, identifying supports for psychosocial well-being is essential [7,23,46,47]. This study found that all three physical activity types—organized sport, unstructured physical activity, and land-use recreation—were significantly associated with improved mental health, emotional control, and social well-being. However, organized youth sport was associated with lower perceived scores in mental health and emotional control compared to unstructured physical activity and land-use recreation, a trend that remained when controlling for gender and community type. These findings emphasize the need for a holistic and individualized approach to physical activity promotion, accounting for contextual and psychosocial factors. While unstructured and land-use-based activities may offer unique psychosocial benefits, all forms of physical activity remain important for youth development and should be universally encouraged.

## Figures and Tables

**Table 1:** Psychosocial benefits of each physical activity type.

	Youth Sport		Unstructured PA		Land-Use	
	*M*	*SD*	*M*	*SD*	*M*	*SD*
Mental Health	3.55	1.44	3.80	1.32	3.81	1.32
Emotional Control	3.65	1.20	3.84	1.32	3.83	1.17
Social Well-being	3.91	1.17	3.98	1.12	3.90	1.17

Note. Median for all groups = 4. PA means physical activity.

**Table 2:** Kruskal–Wallis test with Dunn’s test.

	Youth Sport * X Unstructured PA				Youth Sport X * Land-Use				Unstructured PA X * Land-Use			
	*z*	*p*	*p adj^a^*	*r*	*z*	*p*	*p adj^a^*	*r*	*z*	*p*	*p adj^a^*	*r*
Mental Health	4.01	<0.001	<0.001	0.069	3.99	<0.001	<0.001	0.068	0.10	0.462		
Emotional Control	3.74	<0.001	<0.001	0.064	3.67	<0.001	<0.001	0.063	0.03	0.486		
Social Wellbeing	0.99	0.161			−0.56	0.289			−1.54	0.062		

Note. Dunn’s test was not conducted for any non-significant results of Kruskal–Wallis Test. PA means physical activity.

**Table 3:** Psychosocial benefits of each physical activity type, separating by gender.

	Youth Sport		Unstructured PA		Land-Use	
	*M*	*SD*	*M*	*SD*	*M*	*SD*
Male						
Mental Health	3.56	1.46	3.83	1.32	3.82	1.34
Emotional Control	3.73	1.22	3.90	1.17	3.91	1.17
Social Well-being	3.92	1.18	4.01	1.12	3.96	1.17
Female						
Mental Health	3.58	1.39	3.78	1.32	3.78	1.30
Emotional Control	3.50	1.17	3.78	1.09	3.70	1.17
Social Well-being	3.90	1.15	3.96	1.12	3.79	1.17

Note. Median for all groups = 4. PA stands for physical activity.

**Table 4:** Kruskal–Wallis test with Dunn’s test, separating by gender.

	Youth Sport * X Unstructured PA			Youth Sport X * Land-Use			Unstructured PA X * Land-Use		
	*z*	*p*	*p adj^a^*	*z*	*p*	*p adj^a^*	*z*	*p*	*p adj^a^*
Male									
Mental Health	3.34	<0.001	0.001	3.21	<0.001	<0.001	−0.090	0.464	
Emotional Control	2.79	0.003	<0.001	2.88	0.002	<0.001	0.11	0.458	
Social Wellbeing	1.22	0.111		0.76	0.222		−0.44	0.332	
Female									
Mental Health	2.12	0.017	0.050	1.99	0.023	0.070	−0.03	0.488	
Emotional Control	3.60	<0.001	<0.001	2.80	0.003	0.008	−0.67	0.251	
Social Wellbeing	0.61	0.272		−1.59	0.056		−2.33	0.01	0.029

Note. Dunn’s test was not conducted for any non-significant results of Kruskal–Wallis Test. PA stands for physical activity.

**Table 5:** Psychosocial benefits of each physical activity type, separating by community type.

	Youth Sport		Unstructured PA		Land-Use	
	*M*	*SD*	*M*	*SD*	*M*	*SD*
Urban						
Mental Health	3.45	1.56	3.68	1.46	3.77	1.40
Emotional Control	3.68	1.31	3.81	1.25	3.83	1.28
Social Well-being	3.82	1.31	3.88	1.27	3.87	1.28
Suburban						
Mental Health	3.68	1.31	3.92	1.21	3.74	1.32
Emotional Control	3.60	1.12	3.85	1.01	3.77	1.09
Social Well-being	4.01	1.03	4.02	0.93	3.85	1.10
Rural						
Mental Health	3.59	1.34	3.89	1.14	4.03	1.09
Emotional Control	3.67	1.06	3.91	1.06	3.91	1.01
Social Well-being	4.02	0.97	4.13	1.04	4.04	0.99

Note. Median for all groups = 4. PA stands for physical activity.

**Table 6:** Kruskal–Wallis test with Dunn’s test, separating by community type.

	Youth Sport * X Unstructured PA			Youth Sport X * Land-Use			Unstructured PA X * Land-Use		
	*z*	*p*	*p adj^a^*	*z*	*p*	*p adj^a^*	*z*	*p*	*p adj^a^*
Urban									
Mental Health	−2.297	0.011	0.032	−3.111	<0.001	0.003	−0.861	0.195	
Emotional Control	−1.442	0.075		−1.980	0.024	0.072	−0.568	0.285	
Social Wellbeing	−0.740	0.230		−0.698	0.242		0.27	0.489	
Suburban									
Mental Health	−2.745	0.003	0.009	−0.874	0.191		1.809	0.035	0.106
Emotional Control	−2.946	0.002	0.005	−2.091	0.018	0.055	0.771	0.220	
Social Wellbeing	0.450	0.326		2.16	0.015	0.046	1.75	0.034	0.119
Rural									
Mental Health	−2.060	0.020	0.059	−3.171	<0.001	0.002	−1.253	0.105	
Emotional Control	−2.64	0.004	0.012	−2.42	0.008	0.023	0.104	0.459	
Social Wellbeing	−1.64	0.05	0.152	−0.22	0.414		1.404	0.080	

Note. Dunn’s test was not conducted for any non-significant results of Kruskal–Wallis Test. PA stands for physical activity.

## Data Availability

Data are available by contacting the author, Travis Dorsch, at travis.dorsch@usu.edu.

## Abbreviation

The following abbreviations are used in this manuscript:

PA	Physical Activity

## Appendix A

**Table A1:** Percentage of Respondents Responding in Each Likert Category.

	Youth Sport					Unstructured PA					Land-Use				
	1	2	3	4	5	1	2	3	4	5	1	2	3	4	5
Mental Health	16.1%	8.43%	14.3%	26.4%	34.8%	10.4%	7.95%	13.1%	28.3%	40.3%	10.4%	7.00%	15.6%	25.8%	41.3%
Emotional Control	7.99%	9.22%	20.2%	34.6%	28.0%	6.46%	4.23%	21.6%	33.9%	33.8%	7.47%	5.51%	17.3%	36.4%	33.3%
Social Well-being	6.67%	6.67%	12.6%	36.3%	37.8%	5.55%	4.97%	15.7%	33.8%	40.0%	6.35%	6.44%	16.7%	32.3%	38.2%

## References

[1] Eime R.M., Young J.A., Harvey J.T., Charity M.J., Payne W.R. (2013). A systematic review of the psychological and social benefits of participation in sport for children and adolescents: Informing development of a conceptual model of health through sport. Int. J. Behav. Nutr. Phys. Act..

[2] Kohl H., Cook H., Committee on Physical Activity and Physical Education in the School Environment (2013). Physical Activity and Physical Education: Relationship to Growth, Development, and Health. Educating the Student Body: Taking Physical Activity and Physical Education to School.

[3] U.S. Department of Health and Human Services (2018). Physical Activity Guidelines for Americans.

[4] Merlo C.L., Jones S.E., Michael S.L., Chen T.J., Sliwa S.A., Lee S.H., Brener N.D., Lee S.M., Park S. (2019). Dietary and Physical Activity Behaviors Among High School Students—Youth Risk Behavior Survey, United States. https://nccd.cdc.gov/youthonline/App/Default.aspx.

[5] Eime R.M., Payne W.R., Casey M.M., Harvey J.T. (2010). Transition in participation in sport and unstructured physical activity for rural living adolescent girls. Health Educ. Res..

[6] Mendoza-Vasconez A.S., Linke S., Muñoz M., Pekmezi D., Ainsworth C., Cano M., Williams V., Marcus B.H., Larsen B.A. (2016). Promoting Physical Activity among Underserved Populations. Curr. Sports Med. Rep..

[7] McGorry P., Gunasiri H., Mei C., Rice S., Gao C.X. (2025). The youth mental health crisis: Analysis and solutions. Front. Psychiatry.

[8] Kharel M., Sakamoto J.L., Carandang R.R., Ulambayar S., Shibanuma A., Yarotskaya E., Basargina M., Jimba M. (2022). Impact of COVID-19 pandemic lockdown on movement behaviours of children and adolescents: A systematic review. BMJ Glob. Health.

[9] Do B., Kirkland C., Besenyi G.M., Smock C.M.P.H., Lanza K. (2022). Youth physical activity and the COVID-19 pandemic: A systematic review. Prev. Med. Rep..

[10] Dorsch T.E., Blazo J.A., Arthur-Banning S.G., Anderson-Butcher D., Jayanthi N., Hardiman A., Farrey T., Solomon J., Lerner J.B. (2021). National Trends in Youth Sport during the COVID-19 Pandemic: Understanding American Parents’ Perceptions and Perspectives. J. Sport Behav..

[11] Larouche R., Kleinfeld M., Rodriguez U.C., Hatten C., Hecker V., Scott D.R., Brown L.M., Onyeso O.K., Sadia F., Shimamura H. (2023). Determinants of Outdoor Time in Children and Youth: A Systematic Review of Longitudinal and Intervention Studies. Int. J. Environ. Res. Public Health.

[12] Whitley M.A., Smith A.L., Dorsch T.E., Bowers M.T. (2021). Reenvisioning Postpandemic Youth Sport to Meet Young People’s Mental, Emotional, and Social Needs. Transl. J. ACSM.

[13] Zhou T., Zhai X., Wu N., Koriyama S., Wang D., Jin Y., Li W., Sawada S.S., Fan X. (2022). Changes in Physical Fitness during COVID-19 Pandemic Lockdown among Adolescents: A Longitudinal Study. Healthcare.

[14] Wen M., Zhang X., Harris C.D., Holt J.B., Croft J.B. (2013). Spatial disparities in the distribution of parks and green spaces in the USA. Ann. Behav. Med..

[15] Caspersen C.J., Powell K.E., Christenson G.M. (1985). Physical Activity, Exercise, and Physical Fitness: Definitions and Distinctions for Health-Related Research Synopsis. Public Health Rep..

[16] Black L.I., Terlizzi E.P., Vahratian A. Key findings Data from the National Health Interview Survey. https://www.cdc.gov/nchs/products/index.htm.

[17] Hulteen R.M., Smith J.J., Morgan P.J., Barnett L.M., Hallal P.C., Colyvas K., Lubans D.R. (2017). Global participation in sport and leisure-time physical activities: A systematic review and meta-analysis. Prev. Med..

[18] Merkel D. (2013). Youth sport: Positive and negative impact on young athletes. Open Access J. Sports Med..

[19] Eigenschenk B., Thomann A., McClure M., Davies L., Gregory M., Dettweiler U., Inglés E. (2019). Benefits of outdoor sports for society. A systematic literature review and reflections on evidence. Int. J. Environ. Res. Public Health.

[20] Appleby K.M., Foster E., Roper E. (2013). Gender and Sport Participation. Gender Relations in Sport.

[21] Fleming D.J.M., Dorsch T.E., Serang S., Hardiman A.L., Blazo J.A., Farrey T., Lerner J.B., Solomon J. (2023). The association of families’ socioeconomic and demographic characteristics with parents’ perceived barriers to returning to youth sport following the COVID-19 pandemic. Psychol. Sport Exerc..

[22] Hardiman A.L., Fleming D.J.M., Dorsch T.E., Blazo J.A., Farrey T., Lerner J.B., Solomon J. (2024). Youth sport during the COVID-19 pandemic: The influence of race and affluence on parents’ perspectives of youth participation. Soc. Sci. Humanit. Open.

[23] Kass P., Morrison T.E. (2023). The Impact of COVID-19 Restrictions on Youth Athlete Mental Health: A Narrative Review. Curr. Psychiatry Rep..

[24] Lee R.L.T., Lane S., Brown G., Leung C., Kwok S.W.H., Chan S.W.C. (2020). Systematic review of the impact of unstructured play interventions to improve young children’s physical, social, and emotional wellbeing. Nurs. Health Sci..

[25] Kinder C.J., Gaudreault K.L., Simonton K. (2020). Structured and Unstructured Contexts in Physical Education: Promoting Activity, Learning and Motivation. J. Phys. Educ. Recreat. Dance.

[26] Subramanian S.K., Sharma V.K., Arunachalam V., Radhakrishnan K., Ramamurthy S. (2015). Effect of structured and unstructured physical activity training on cognitive functions in adolescents—A randomized control trial. J. Clin. Diagn. Res..

[27] Brockman R., Jago R., Fox K.R., Thompson J.L., Cartwright K., Page A.S. (2009). “Get off the sofa and go and play”: Family and socioeconomic influences on the physical activity of 10–11 year old children. BMC Public Health.

[28] Hagan J.F., Shaw J.S., Duncan P.M., American Academy of Pediatrics (2017). Promoting Physical Activity. Bright Futures: Guidelines for Health Supervision of Infants, Children and Adolescents.

[29] Coen S.E., Mitchell C.A., Tillmann S., Gilliland J.A. (2019). “I like the ‘outernet’ stuff:” Girls’ perspectives on physical activity and their environments. Qual. Res. Sport Exerc. Health.

[30] Nathan A., George P., Ng M., Wenden E., Bai P., Phiri Z., Christian H. (2021). Impact of COVID-19 restrictions on Western Australian children’s physical activity and screen time. Int. J. Environ. Res. Public Health.

[31] Pelletier C.A., Cornish K., Sanders C. (2021). Children’s independent mobility and physical activity during the COVID-19 pandemic: A qualitative study with families. Int. J. Environ. Res. Public Health.

[32] Noseworthy M., Peddie L., Buckler E.J., Park F., Pham M., Pratt S., Singh A., Puterman E., Liu-Ambrose T. (2023). The Effects of Outdoor versus Indoor Exercise on Psychological Health, Physical Health, and Physical Activity Behaviour: A Systematic Review of Longitudinal Trials. Int. J. Environ. Res. Public Health.

[33] Thompson Coon J., Boddy K., Stein K., Whear R., Barton J., Depledge M.H. (2011). Does participating in physical activity in outdoor natural environments have a greater effect on physical and mental wellbeing than physical activity indoors? A systematic review. Environ. Sci. Technol..

[34] Bailey A.W., Kang H.-K. (2022). Walking and Sitting Outdoors: Which Is Better for Cognitive Performance and Mental States?. Int. J. Environ. Res. Public Health.

[35] Mann J., Gray T., Truong S., Sahlberg P., Bentsen P., Passy R., Ho S., Ward K., Cowper R. (2021). A Systematic Review Protocol to Identify the Key Benefits and Efficacy of Nature-Based Learning in Outdoor Educational Settings. Int. J. Environ. Res. Public Health.

[36] McCurdy L.E., Winterbottom K.E., Mehta S.S., Roberts J.R. (2010). Using nature and outdoor activity to improve children’s health. Curr. Probl. Pediatr. Adolesc. Health Care.

[37] Himschoot E., Lloyd J., Reuben A. (2020). Improving Child & Adolescent Mental Health Through Outdoor Programming: Engaging the Land Conservation Community.

[38] Outdoor Industry Association (2022). 2022 Outdoor Participation Trends Report.

[39] Rizzolo J.B., Delie J., Carlson S.C., Dietsch A.M. (2023). Gender differences in wildlife-dependent recreation on public lands. Front. Conserv. Sci..

[40] Kaczynski A.T., Henderson K.A. (2007). Environmental correlates of physical activity: A review of evidence about parks and recreation. Leis. Sci..

[41] Diaz-Perez F., García-González C., Fyall A., Fu X., Deel G., Fernandez-Hdez C. (2025). The altered perceptions of visitors to national parks: A comparison between a pre- and post-COVID-19 periods. Soc. Sci. Humanit. Open.

[42] Jackson S.B., Stevenson K.T., Larson L.R., Peterson M.N., Seekamp E. (2021). Outdoor Activity Participation Improves Adolescents’ Mental Health and Well-Being during the COVID-19 Pandemic. Int. J. Environ. Res. Public Health.

[43] Biddle S.J.H., Ciaccioni S., Thomas G., Vergeer I. (2019). Physical activity and mental health in children and adolescents: An updated review of reviews and an analysis of causality. Psychol. Sport Exerc..

[44] Hoza B., Smith A.L., Shoulberg E.K., Linnea K.S., Dorsch T.E., Blazo J.A., Alerding C.M., McCabe G.P. (2015). A randomized trial examining the effects of aerobic physical activity on attention-deficit/hyperactivity disorder symptoms in young children. J. Abnorm. Child Psychol..

[45] Herrman H., Saxena S., Moodie R., World Health Organization, Victorian Health Promotion Foundation, University of Melbourne (2005). Promoting Mental Health: Concepts, Emerging Evidence, Practice.

[46] Bitsko R.H., Holbrook J.R., Ghandour R.M., Blumberg S.J., Visser S.N., Perou R., Walkup J.T. (2018). Epidemiology and impact of health care provider-diagnosed anxiety and depression among US children. J. Dev. Behav. Pediatr..

[47] Meade J. (2021). Mental Health Effects of the COVID-19 Pandemic on Children and Adolescents: A Review of the Current Research. Pediatr. Clin. N. Am..

[48] Physical Activity Guidelines Advisory Committee (2018). 2018 Physical Activity Guidelines Advisory Committee Scientific Report.

[49] Donnelly J.E., Hillman C.H., Castelli D., Etnier J.L., Lee S., Tomporowski P., Lambourne K., Szabo-Reed A.N. (2016). Physical activity, fitness, cognitive function, and academic achievement in children: A systematic review. Med. Sci. Sports Exerc..

[50] Suarez-Manzano S., Ruiz-Ariza A., De La Torre-Cruz M., Martínez-López E.J. (2018). Acute and chronic effect of physical activity on cognition and behaviour in young people with ADHD: A systematic review of intervention studies. Res. Dev. Disabil..

[51] Gross J.J. (2002). Emotion regulation: Affective, cognitive, and social consequences. Psychophysiology.

[52] Bernstein E.E., McNally R.J. (2018). Exercise as a buffer against difficulties with emotion regulation: A pathway to emotional wellbeing. Behav. Res. Ther..

[53] Edwards M.K., Rhodes R.E., Mann J.R., Loprinzi P.D. (2018). Effects of acute aerobic exercise or meditation on emotional regulation. Physiol. Behav..

[54] Puett R., Teas J., España-Romero V., Artero E.G., Lee D.C., Baruth M., Sui X., Montresor-López J., Blair S.N. (2014). Physical activity: Does environment make a difference for tension, stress, emotional outlook, and perceptions of health status?. J. Phys. Act. Health.

[55] Belliveau J., Soucy K.I., Yakovenko I. (2022). The validity of qualtrics panel data for research on video gaming and gaming disorder. Exp. Clin. Psychopharmacol..

[56] Shapiro S.S., Wilk M.B. (1965). An analysis of variance test for normality (complete samples). Biometrika.

[57] McKight P.E., Najab J., Weiner I.B., Craighead W.E. (2010). Kruskal-Wallis Test. The Corsini Encyclopedia of Psychology.

[58] Dinno A. (2015). Nonparametric pairwise multiple comparisons in independent groups using Dunn’s test. Stata J..

[59] R Core Team (2024). R: A Language and Environment for Statistical Computing.

